# Protein‐enriched soup and weekly exercise improve muscle health: A randomized trial in mid‐to‐old age with inadequate protein intake

**DOI:** 10.1002/jcsm.13481

**Published:** 2024-04-20

**Authors:** Li‐Ning Peng, Ming‐Hsien Lin, Sung‐Hua Tseng, Ko‐Han Yen, Huei‐Fang Lee, Fei‐Yuan Hsiao, Liang‐Kung Chen

**Affiliations:** ^1^ Center for Geriatrics and Gerontology Taipei Veterans General Hospital Taipei Taiwan; ^2^ Center for Healthy Longevity and Aging Sciences National Yang Ming Chiao Tung University Taipei Taiwan; ^3^ Graduate Institute of Clinical Pharmacy, College of Medicine National Taiwan University Taipei Taiwan; ^4^ School of Pharmacy, College of Medicine National Taiwan University Taipei Taiwan; ^5^ Department of Pharmacy National Taiwan University Hospital Taipei Taiwan; ^6^ Taipei Municipal Gan‐Dau Hospital (Managed by Taipei Veterans General Hospital) Taipei Taiwan

**Keywords:** Metabolism, Muscle health, Protein intake

## Abstract

**Background:**

Prior research has highlighted the synergistic impact of protein supplementation on muscle function post‐exercise in adults; however, evidence supporting the combined effects were less robust and inconsistent on those with protein insufficiency. This investigation aims to explore efficacy of protein‐enriched soup coupled with exercise on muscle health and metabolism in middle‐aged and older adults with suboptimal protein intake.

**Methods:**

An open‐label, 12‐week, randomized controlled trial involving participants with insufficient protein intake (<1.0 g/kg/day) was done. The intervention group consumed protein‐enriched soup (24–30 g protein daily) and 1‐h weekly exercise, while controls received health education. Assessments included laboratory tests, functional assessments, and body composition.

**Results:**

In this trial, 97 out of 100 randomized participants (mean age: 64.65 ± 4.84 years, 81.8% female) completed the study (47 in intervention group and 50 in control group). Compared results of baselines, at 1 and 3 months of intervention, significant improvements in waist circumference (83.48 ± 10.22 vs. 82.5 ± 9.88 vs. 82.37 ± 9.42 cm, *P* for trend = 0.046), 6‐min walking distance (525.65 ± 58.46 vs. 534.47 ± 51.87 vs. 552.02 ± 57.66 m, *P* for trend = 0.001), five‐time sit‐to‐stand time (7.63 ± 1.63 vs. 6.81 ± 1.8 vs. 6.4 ± 1.42 s, *P* for trend <0.001), grip strength (26.74 ± 6.54 vs. 27.53 ± 6.99 vs. 28.52 ± 7.09 kg, *P* for trend <0.001), and MNA score (26.8 ± 2.14 vs. 27.73 ± 1.74 vs. 27.55 ± 1.72, *P* for trend <0.001) were discerned within the intervention group. The intervention demonstrated a significant reduction in serum triglyceride (105.32 ± 49.84 vs. 101.36 ± 42.58 vs. 93.43 ± 41.49 mg/dL, *P* for trend = 0.023), increased HDL‐C (60.04 ± 16.21 vs. 60 ± 17.37 vs. 62.55 ± 18.27 mg/dL, *P* for trend = 0.02), and DHEA‐S levels (97.11 ± 54.39 vs. 103.39 ± 56.75 vs. 106.83 ± 60.56 μg/dL, *P* for trend = 0.002). Serum myostatin did not differ in both groups, but serum leptin levels significantly increased (9118.88 ± 5811.68 vs. 11508.97 ± 7151.08 vs. 11220.80 ± 7190.71 pg/mL, *P* for trend = 0.016) in controls. The intervention group showed greater improvements in 6 min walking distance (β = 0.71, 95% CI: 6.88 to 40.79, *P* = 0.006), five‐time sit‐to‐stand test (β = −0.87, 95% CI: −1.59 to −0.15, *P* = 0.017), MNA score (β = 0.96, 95% CI: 0.20 to 1.71, *P* = 0.013), serum triglycerides (β = −15.01, 95% CI: −27.83 to −2.20, *P* = 0.022), LDL‐C (β = −9.23, 95% CI: −16.98 to −1.47, *P* = 0.020), and DHEA‐S levels (β = 9.98, 95% CI: 0.45 to 19.51, *P* = 0.04) than controls.

**Conclusions:**

Protein‐enriched soup with weekly exercise over 12 weeks significantly improved physical performance, lipid profile, and DHEA‐S levels among middle‐aged and older adults with inadequate protein intake, while studies assessing long‐term benefits of the intervention are needed.

## Introduction

Given the global trend of population aging, there is a shared value to prolong the healthy life expectancy, leading the World Health Organization to define healthy aging as the process of developing and maintaining functional ability over time to secure well‐being in later stages of life.^1^ However, maintaining functional ability in aging individuals necessitates a comprehensive strategy that considers the interplay of multiple factors, including disturbances in homeostasis, reduced physiological reserves, compromised organ function, and metabolic alterations.^2^ Preserving muscle health is crucial to promote well‐being of older adults, as age‐related changes in skeletal muscle morphology and physiology can lead to reductions in muscle mass, strength, and endurance, resulting in functional declines, increased risk of falls, decreased mobility, and loss of autonomy.^3^ As such, delineating the pathophysiological mechanisms underlying muscle protein breakdown and the onset of sarcopenia is imperative to preclude the emergence of this debilitating condition and the associated functional limitations.

A holistic approach encompassing regular exercise, optimal nutrition, and hormone balance is important in preserving muscle health and preventing age‐related functional decline, as adequate nutrition is essential for the growth, repair, and maintenance of skeletal muscles.^4^ The timely and adequate provision of nutrition is critical in mitigating the risks of falls, hospitalizations, and long‐term care admissions, minimizing the exacerbation of chronic conditions,^5^, ^6^ and addressing potential complications associated with malnutrition, including physical and cognitive declines, reduced immunity, depression, and impaired quality of life.^7^, ^8^ The importance of protein as a vital nutrient for preserving muscle health in older adults is highlighted by a substantial body of literature, considering the age‐related increase in muscle loss.^9^, ^10^ The Health, Aging, and Body Composition (Health‐ABC) Study provides compelling evidence supporting the protective effects of protein intake on age‐related muscle loss and the maintenance of lean mass, with older adults in the highest quintiles of protein consumption showing a significantly lower decline in lean mass.^11^ Some studies indicate that higher amino acid and protein intake is associated with a lower incidence of frailty among older adults,^12^ while sufficient protein intake support reduces the risk of falls and disability and improves overall quality of life.^13^, ^14^, ^15^ In addition, protein supplementation may influence the regulation of certain cytokines like myostatin and leptin, which are also body composition, muscle functionality, and the overall health among the middle‐aged and senior populations.^16^, ^17^, ^18^

According to the PROT‐AGE study group, individuals aged over 65 years should aim for a daily protein intake of 1.0 to 1.2 g/kg of body weight to maintain and restore lean body mass and function, while those with acute or chronic conditions may benefit from a higher intake of 1.2–1.5 grams of protein per kilogram of body weight.^19^ The Asian Working Group for Sarcopenia (AWGS) suggests a minimum protein intake of 1.0 g/kg/day for healthy older adults, while individuals at risk for sarcopenia or frailty should target an intake of 1.2 g/kg/day.^20^, ^21^ Achieving the recommended daily protein intake should primarily be done through a well‐balanced diet; however, protein supplementation could be a viable option for those unable to meet the target through dietary sources.^22^ Despite the abovementioned guidelines, 23–27% of community‐dwelling older adults have insufficient protein intake (<1.0 g/kg/day), as revealed by a recent systematic review.^23^ The palatability of oral nutritional supplements (ONS) is often suboptimal for at‐risk individuals, particularly older individuals, and lower dairy intake in the Asian population is attributed to a high prevalence of lactose intolerance despite evidence of its benefits for muscle health.^24^ Building upon our previous clinical trial showing no beneficial clinical outcomes (physical performance and metabolism) with high‐dose protein supplementation,^25^ the present trial investigates the impact of a fixed‐dose protein‐enriched soup plus exercise on muscle health, physical performance, and associated biomarkers in community‐dwelling middle‐aged and older adults with inadequate protein intake.

## Methods

### Study design and participants

This study is an open‐label, randomized controlled trial conducted from 15 June 2022 to 1 January 2023 in Taipei City. Participants were recruited from the community. Its objective is to investigate the effects of protein‐enriched soup in conjunction with once weekly exercise intervention on muscle health and metabolism of community‐dwelling middle‐aged and older adults. We used physical function encompassing five times chair‐stand test, 6‐min walking distance, gait speed, and handgrip strength as primary outcomes.

All the participants for the trial were community‐dwelling individuals aged 50–75 years, at least one fall event within the past year, providing informed consent and agreeing to comply with the study protocol were eligible to join this trial. Those who with a daily protein intake below 1.0 g/kg/day were eligible for inclusion in this study. Each participant had documented their dietary intake for the day preceding screening, and professional dieticians assessed the quantity of protein consumed by each participant. Those who were unable to follow the study design, had a 6‐m usual gait speed of <0.3 m/s, suffered functional impairment due to extremity fractures or severe arthritis, were undergoing cancer treatment, had a history of gouty arthritis or severe renal insufficiency (estimated glomerular filtration rate <30 mL/min/1.73 m^2^), suffered from severe visual or hearing impairment, received hormone therapy within the last 3 months or intended to during the study period, had clinical conditions that were unsuitable for the study, such as uncontrolled cardiopulmonary disease or uncontrolled mental illness, or had neurological diseases such as stroke, parkinsonism, or peripheral neuropathy with intermittent claudication were excluded, as were those with contraindications for bioimpedance analysis (BIA) examination. Sample size calculation was based on physical function from a previous meta‐analysis, which showed significant improvement in handgrip strength and SPPB, the effect size were 0.29 and 0.32, respectively.^18^ Hence, we assumed with the effective size of 0.3, and at least 100 participants is needed to achieve a discriminatory power of 80% at the two‐tail α significance level of 5% and considering an estimated dropout rate of 20%.

This study aimed to recruit 100 eligible participants who were randomly assigned to either the intervention or control group at a 1:1 ratio, with the intervention group receiving a daily serving of protein‐enriched soup containing 24–30 g of protein and weekly 1 h group exercise, which encompassed moderate aerobic exercise and resistant exercise. Participants in the control group underwent a 1h health education session upon enrolment in the study, focusing on the basic knowledge about proper nutrition and exercise for older adults. The randomization sequence was generated by EXCEL and sealed in an envelope until the participant agreed to participate in the trial and follow the protocol, which involved three visits (at week 0, 4, and 12) at the clinical site. During the initial and subsequent visits, all participants underwent a functional assessment, laboratory tests, and body composition analysis.

The study protocol was approved by the Institutional Review Board of Yang Ming Chiao Tung University (YM109172F and YM111058F), and was prospectively registered at ClinicalTrial.gov (NCT05828134). All participants provided informed consent prior to participating in the study procedures.

### Demographic characteristics and laboratory data

The study employed research nurses to collect demographic and anthropometric data, as well as medical history information from all participants. Tobacco usage was categorized as either current smoking or non‐smoking, while status of alcohol consumption was divided into drinking or non‐drinking habits. Peripheral venous blood samples were obtained from all participants after a 10 h overnight fast, and various biochemical parameters were measured, including serum albumin, creatinine, alanine aminotransferase, total cholesterol, triglyceride, high‐density lipoprotein cholesterol (HDL‐C), low‐density lipoprotein cholesterol (LDL‐C), uric acid, fasting glucose, dehydroepiandrosterone sulfate (DHEA‐S), insulin‐like growth factor‐1 (IGF‐1), homocysteine, high‐sensitive CRP (hs‐CRP), and vitamin D3. The homeostasis model assessment of insulin resistance (HOMA‐IR) was calculated as fasting glucose (mg/dL) × fasting insulin (mIU/L)/405. The study also tested whole blood glycated haemoglobin (HbA1c) using high‐performance liquid chromatography (Bio‐Rad D‐100 System, Bio‐Rad, USA). Additionally, serum levels of myostatin and leptin were measured by GDF/Myostatin Quantikine enzyme‐linked immunosorbent assay (ELISA) and Human Total Adiponectin/Acro30, DRP300; Human Leptin, DLP00 purchased from R&D Systems, Inc. (Minneapolis, MN, USA) following the manufacturer's instructions.

### Functional assessment and body composition

**Figure 1:**
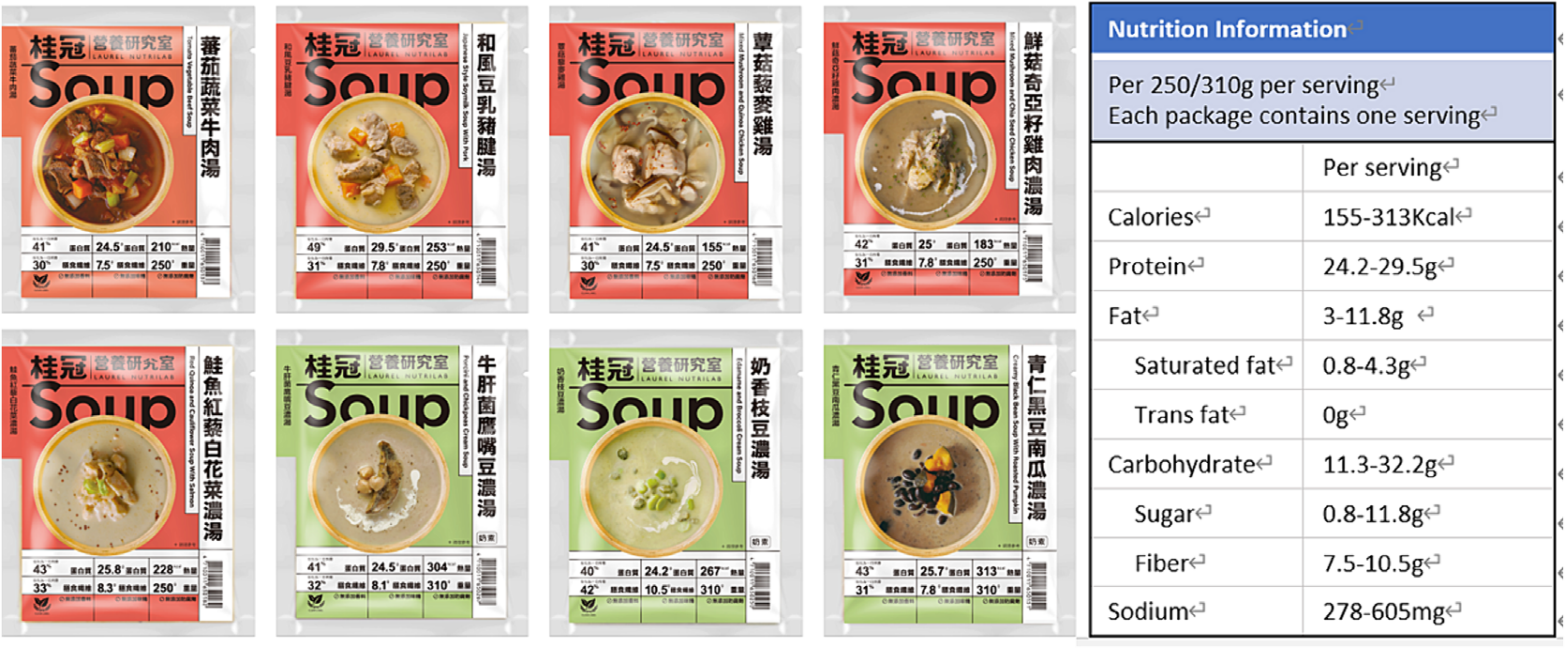
Nutritional information of the protein enriched soups.

The study participants underwent a thorough functional assessment, which was conducted by well‐trained research nurses. The assessment involved several aspects, including cognitive function [Montreal Cognitive Assessment (MoCA)],^26^ mood status [Center for Epidemiologic Studies Depression Scale (CES‐D)],^27^ nutritional status [Mini‐Nutritional Assessment (MNA)],^28^ physical activity [International Physical Activity Questionnaire (IPAQ)],^29^ and the severity of underlying medical conditions [Charlson co‐morbidity index (CCI)].^30^ Handgrip strength was measured using a dynamometer (Smedley's Dynamometer; TTM, Tokyo, Japan) of the dominant hand, while usual gait speed and muscle endurance were evaluated through the 6 m walking test and 6 min walking distance, respectively. The quality of life was evaluated using the 36‐item Short Form Survey (SF‐36).^31^ Additionally, the participants' body composition was captured by bioelectrical impedance analysis (BIA) using the Inbody S10 device by Biospace, USA, which provided data on the relative appendicular skeletal muscle mass (RASM) and total body fat percentage (Figure 1).

### Statistical analysis

In the present study, the analytical approach employed was an intention‐to‐treat design, which included data of all participants for analysis. Categorical data were expressed as percentages while continuous data were expressed as means with their corresponding standard deviation. For comparisons of categorical data, either the chi‐squared or Fisher's exact test was employed, while for continuous variables, the Student's *t*‐test or Mann–Whitney *U* test was used. Interval changes in participant variables within groups were examined using paired *t*‐tests. Repeated measures ANOVA was used to analyse the changes in variables over time. A generalized linear regression model was employed to assess the intervention effects across different groups. All statistical analyses were conducted using the IBM SPSS statistics Version 24.0 for Microsoft Window XP (SPSS Inc., Chicago, IL, USA) and SAS statistical package, Version 9.4 (SAS Institute, Inc., Cary, NC, USA). A two‐tailed *P*‐value of <0.05 was deemed statistically significant.

## Results

**Figure 2:**
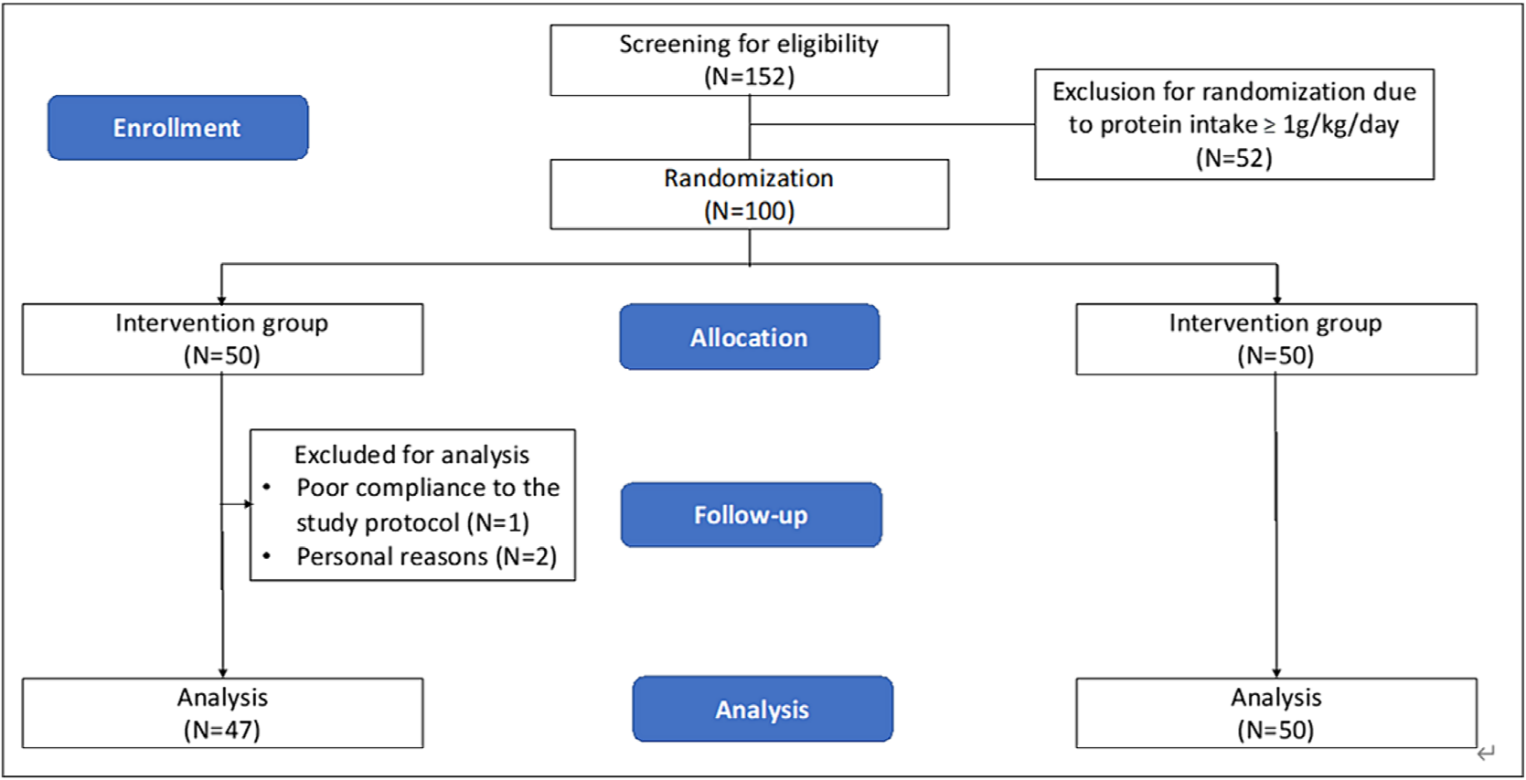
Flowchart of the randomized controlled trial.

The study underwent a rigorous screening process, whereby 152 prospective participants were initially assessed, but 52 were excluded prior to randomization. Consequently, a total of 100 eligible participants were randomly assigned in a 1:1 ratio into two groups. The mean age of all participants was 64.66 ± 4.84 years, with 81.8% being female. Notably, the participants' average daily protein intake before the study was 0.80 ± 0.17 g/kg. However, three participants in the interventional group did not complete the trial, leaving a total of 97 participants (47 in the intervention group and 50 in the control group) (Figure 2). Reasons for the withdrawing from the trial were not intervention‐related but due to personal health issues.

Table 1 provides a summary of the baseline characteristics of all participants, including functional assessments, anthropometric measurements, and laboratory results, and compares the two groups. No significant differences were observed between the intervention and control groups in terms of baseline demographic characteristics, functional assessments, anthropometric measurements, or laboratory results. The baseline nutritional status was assessed and compared between both groups as shown in Data S1, indicating no significant differences across the variables measured by MNA. In Table 2, a detailed comparison of the within‐group effects of the intervention on the participants is presented, encompassing the data from before the study as well as the first and third months after the initiation of the study. In the intervention group, a progressive and significant reduction in waist circumference was observed, evident both before the intervention and at 1 and 3 months post‐intervention (83.48 ± 10.22 vs. 82.5 ± 9.88 vs. 82.37 ± 9.42 cm, *P* for trend = 0.046). Moreover, the improvement in functional parameters, such as 6‐min walking distance (525.65 ± 58.46 vs. 534.47 ± 51.87 vs. 552.02 ± 57.66 m, *P* for trend = 0.001), time of 5‐time sit‐to‐stand (7.63 ± 1.63 vs. 6.81 ± 1.80 vs. 6.4 ± 1.42 s, *P* for trend <0.001), handgrip strength (26.74 ± 6.54 vs. 27.53 ± 6.99 vs. 28.52 ± 7.09 kg, *P* for trend <0.001), and MNA score (26.8 ± 2.14 vs. 27.73 ± 1.74 vs. 27.55 ± 1.72, *P* for trend <0.001) were also observed. Additionally, the intervention group exhibited significant decrease in serum levels of triglycerides (105.32 ± 49.84 vs. 101.36 ± 42.58 vs. 93.43 ± 41.49 mg/dL, *P* for trend = 0.023) and increases in HDL‐C (60.04 ± 16.21 vs. 60 ± 17.37 vs. 62.55 ± 18.27 mg/dL, *P* for trend = 0.02) and DHEA‐S levels (97.11 ± 54.39 vs. 103.39 ± 56.75 vs. 106.83 ± 60.56 μg/dL, *P* for trend = 0.002). On the other hand, the control group exhibited a significant increase in handgrip strength (24.74 ± 6.9 vs. 24.58 ± 7.5 vs. 25.9 ± 7.05 kg, *P* for trend = 0.002), serum leptin level (9118.88 ± 5811.68 vs. 11508.97 ± 7151.08 vs. 11220.80 ± 7190.71 mg/dL, *P* for trend = 0.007) levels and marginal increase of LDL‐C levels (110.92 ± 22.31 vs. 115.97 ± 30.25 vs. 118.38 ± 22.19, *P* for trend = 0.065).

**Table 1:** Demographic characteristics of study group versus control group before interventions

Data show mean ± standard deviation or %	Intervention group	Placebo group	*P* value
	(*N* = 47)	(*N* = 50)	
Demographic data			
Age	65.09 ± 5.13	64.22 ± 4.66	0.386
Female (%)	76.60%	86.00%	0.299
Education years	14.11 ± 2.31	13.46 ± 2.92	0.231
Smoking (%)	0.0%	2.0%	0.330
Alcohol consumption (%)	19.1%	20.0%	0.766
CCI	0.57 ± 0.9	0.68 ± 0.79	0.542
Body composition and functional assessment			
BMI	22.86 ± 3.42	22.73 ± 2.77	0.830
Waist circumference (cm)	83.48 ± 10.22	82.66 ± 8.48	0.666
Hip circumference (cm)	94.82 ± 6.3	94.75 ± 4.76	0.950
RASM (kg/m^2^)	6.49 ± 0.94	6.26 ± 0.7	0.164
Male	7.76 ± 0.71	7.39 ± 0.63	0.293
Female	6.11 ± 0.61	6.06 ± 0.50	0.717
Gait speed (m/s)	1.75 ± 0.23	1.7 ± 0.32	0.465
6‐min walking distance (m)	524.36 ± 58.49	532.34 ± 66.61	0.533
Handgrip strength (kg)	26.74 ± 6.54	24.74 ± 6.9	0.145
5‐time chair‐stand test (sec)	7.62 ± 1.61	8.15 ± 2.06	0.164
IPAQ (kcal)	1689.75 ± 1304.55	1,340 ± 876.66	0.122
MNA	26.8 ± 2.14	27.26 ± 1.76	0.247
MoCA	27.64 ± 1.88	27.12 ± 2.48	0.251
CESD	2.62 ± 5.4	3.44 ± 6.15	0.487
Laboratory data			
Albumin (mg/dL)	4.45 ± 0.22	4.44 ± 0.14	0.780
ALT (IU/mL)	24.81 ± 17.12	22.02 ± 7.15	0.293
Creatinine (mg/dL)	0.76 ± 0.13	0.74 ± 0.18	0.581
Fasting glucose (mg/dL)	92.81 ± 16.6	99.28 ± 31.33	0.211
HOMA‐IR	1.72 ± 1.15	1.93 ± 1.41	0.427
Total cholesterol (mg/dL)	196.26 ± 31.83	197.76 ± 28.15	0.805
Triglyceride (mg/dL)	105.32 ± 49.84	104.52 ± 44.07	0.933
HDL (mg/dL)	60.04 ± 16.21	61.94 ± 14.9	0.550
LDL (mg/dL)	114.94 ± 26.33	110.92 ± 22.31	0.419
CRP (mg/dL)	0.07 ± 0.1	0.16 ± 0.44	0.150
DHEA‐S (μg/dL)	97.11 ± 54.39	100.19 ± 45.8	0.763
Leptin (pg/mL)	7267.64 ± 4668.03	9163.26 ± 5674.23	0.131
WBC (10^3^/uL)	4.91 ± 1.17	5.19 ± 1.2	0.249
Hb (g/dL)	13.83 ± 1.39	13.43 ± 1.01	0.103
Myostatin (pg/mL)	4281.87 ± 1549.26	4311.56 ± 1230.72	0.917

ALT, alanine transaminase; BMI, body mass index; CCI, Charlson Co‐morbidity Index; CES‐D, Center for Epidemiological Studies Depression Scale; CRP, C‐reaction protein; DHEA‐S, dehydroepiandrosterone sulfate; Hb, haemoglobin; HDL, high‐density lipoprotein cholesterol; HOMA‐IR, homeostasis model assessment of insulin resistance; IPAQ, The International Physical Activity Questionnaire; LDL, low‐density lipoprotein cholesterol; MNA, Mini‐Nutritional Assessment; MoCA, Montreal Cognitive Assessment; RASM, relative skeletal muscle index; WBC, white blood cell.

**Table 2:** Within‐group comparison in intervention group and placebo group during 3 month intervention period

	Intervention group (*N* = 47)				Placebo group (*N* = 50)			
	Baseline	1 months	3 months	*P* for trend	Baseline	1 months	3 months	*P* for trend
Body composition and functional assessment								
BMI	22.86 ± 3.42	22.86 ± 3.3	22.9 ± 3.39	0.684	22.73 ± 2.77	22.8 ± 2.67	22.64 ± 2.82	0.763
Waist circumference (cm)	83.48 ± 10.22	82.5 ± 9.88	82.37 ± 9.42	0.046	82.66 ± 8.48	83.12 ± 8.38	83.08 ± 8.78	0.434
Hip circumference (cm)	94.82 ± 6.3	94.13 ± 5.92	94.41 ± 5.53	0.086	94.75 ± 4.76	94.07 ± 4.97	94.23 ± 4.89	0.591
RASM (kg/m2)	6.49 ± 0.94	6.5 ± 0.9	6.49 ± 0.95	0.941	6.26 ± 0.7	6.19 ± 0.72	6.29 ± 0.7	0.776
Male	7.76 ± 0.71	7.70 ± 0.72	7.77 ± 0.68	0.598	7.39 ± 0.63	7.77 ± 0.38	7.28 ± 0.72	0.865
Female	6.11 ± 0.61	6.13 ± 0.57	6.10 ± 0.61	0.510	6.06 ± 0.50	6.00 ± 0.49	6.12 ± 0.54	0.759
Gait speed (m/s)	1.74 ± 0.23	1.8 ± 0.19	1.81 ± 0.27	0.057	1.7 ± 0.32	1.69 ± 0.26	1.73 ± 0.28	0.544
6‐min walking distance (m)	525.65 ± 58.46	534.47 ± 51.87	552.02 ± 57.66	0.001	530.04 ± 65.27	529.72 ± 73.52	533.47 ± 70.56	0.607
5 times sit‐to‐stand (sec)	7.63 ± 1.63	6.81 ± 1.8	6.4 ± 1.42	<0.001	8.15 ± 2.06	7.72 ± 2.06	7.81 ± 2.58	0.209
Handgrip strength (kg)	26.74 ± 6.54	27.53 ± 6.99	28.52 ± 7.09	0.001	24.74 ± 6.9	24.58 ± 7.5	25.9 ± 7.05	0.002
IPAQ (Kcal)	1623.65 ± 1236.84	1977.57 ± 1478.12	2066.57 ± 1285.46	0.066	1,340 ± 876.66	1568.55 ± 1146.23	1359.53 ± 1020.06	0.470
MNA	26.8 ± 2.14	27.73 ± 1.74	27.55 ± 1.72	<0.001	27.26 ± 1.76	27.18 ± 1.65	27.06 ± 2.95	0.698
CESD	2.62 ± 5.4	1.62 ± 5.23	1.81 ± 5.73	0.245	3.44 ± 6.15	3.87 ± 8.22	2.26 ± 4.05	0.299
Laboratory data								
Albumin (mg/dL)	4.45 ± 0.22	4.49 ± 0.21	4.5 ± 0.21	0.175	4.44 ± 0.14	4.44 ± 0.23	4.47 ± 0.2	0.366
ALT (IU/mL)	24.81 ± 17.12	24.72 ± 11.1	23.98 ± 10.47	0.641	22.02 ± 7.15	24.46 ± 11.77	23.58 ± 12.41	0.448
Creatinine (mg/dL)	0.76 ± 0.13	0.76 ± 0.13	0.75 ± 0.12	0.752	0.74 ± 0.18	0.73 ± 0.13	0.75 ± 0.16	0.772
Fasting glucose (mg/dL)	92.81 ± 16.6	93.68 ± 13.66	94.96 ± 16.04	0.473	99.28 ± 31.33	97 ± 23.61	100.54 ± 30.62	0.346
HOMA_IR	1.72 ± 1.15	2.15 ± 2.57	1.88 ± 1.46	0.239	1.93 ± 1.41	2.28 ± 2.66	2.32 ± 2.94	0.144
Total cholesterol (mg/dL)	196.26 ± 31.83	194.11 ± 29.61	195.91 ± 35.95	0.727	197.76 ± 28.15	198.69 ± 35.91	205.72 ± 29.65	0.135
Triglyceride (mg/dL)	105.32 ± 49.84	101.36 ± 42.58	93.43 ± 41.49	0.023	104.52 ± 44.07	93.97 ± 32.15	107.64 ± 55.42	0.183
HDL (mg/dL)	60.04 ± 16.21	60 ± 17.37	62.55 ± 18.27	0.020	61.94 ± 14.9	63.54 ± 16.12	62.32 ± 16.05	0.541
LDL (mg/dL)	114.94 ± 26.33	112.3 ± 25.35	113.17 ± 26.97	0.612	110.92 ± 22.31	115.97 ± 30.25	118.38 ± 22.19	0.065
CRP (mg/dL)	0.07 ± 0.1	0.08 ± 0.16	0.09 ± 0.18	0.672	0.16 ± 0.44	0.2 ± 0.67	0.14 ± 0.2	0.384
DHEA‐S (μg/dL)	97.11 ± 54.39	103.39 ± 56.75	106.83 ± 60.56	0.002	100.19 ± 45.8	94.48 ± 42.88	99.93 ± 44.77	0.670
Leptin (pg/mL)	8016.96 ± 5695.92	8610.37 ± 6014.55	8752.98 ± 5779.81	0.174	9118.88 ± 5811.68	11508.97 ± 7151.08	11220.80 ± 7190.71	0.016
WBC (10^3^/uL)	4.91 ± 1.17	4.98 ± 1.04	5.04 ± 1.18	0.654	5.19 ± 1.2	5.54 ± 1.52	5.47 ± 1.62	0.136
Hb (g/dL)	13.83 ± 1.39	13.94 ± 1.26	14.02 ± 1.22	0.111	13.43 ± 1.01	13.43 ± 1.08	13.5 ± 0.97	0.404
Myostatin (pg/mL)	4281.87 ± 1549.26	4388.85 ± 1639.4	4369.63 ± 1678.35	0.846	4311.56 ± 1230.72	4429.23 ± 1554.63	4570.62 ± 1626.39	0.458

ALT, alanine transaminase; BMI, body mass index; CCI, Charlson Co‐morbidity Index; CES‐D, Center for Epidemiological Studies Depression Scale; CRP, C‐reaction protein; DHEA‐S, dehydroepiandrosterone sulfate; Hb, haemoglobin; HDL, high‐density lipoprotein cholesterol; HOMA‐IR, homeostasis model assessment of insulin resistance; IPAQ, The International Physical Activity Questionnaire; LDL, low‐density lipoprotein cholesterol; MNA, Mini‐Nutritional Assessment; MoCA, Montreal Cognitive Assessment; RASM, relative skeletal muscle index; WBC, white blood cell.

Table 3 summarized the analysis using the generalized estimating equation to explore the changes of variables in multiple measurements, which presented the intervention effects that participants in the intervention group had significant improvements compared to the control group in several parameters. Notably, the 6‐min walking distance improved significantly (β = 0.71, 95% CI: 6.88 to 40.79, *P* = 0.006), indicating enhanced physical endurance. The five‐time sit‐to‐stand test showed a significant improvement (β = −0.87, 95% CI: −1.59 to −0.15, *P* = 0.017), reflecting an enhanced physical performance. In addition, the MNA score increased significantly in the intervention group (β = 0.96, 95% CI: 0.20 to 1.71, *P* = 0.013), indicating improved nutritional status. Blood laboratory test results showed a significant reduction in serum triglycerides (β = −15.01, 95% CI: −27.83 to −2.20, *P* = 0.022) and LDL‐C (β = −9.23, 95% CI: −16.98 to −1.47, *P* = 0.020) levels, suggesting an improved lipid profile and metabolism. Moreover, DHEA‐S levels were significantly increased in the intervention group (β = 9.98, 95% CI: 0.45 to 19.51, *P* = 0.04). These findings indicate the significant positive effects of the intervention on multiple health indicators compared to the control group. As a result of the analysis model, serum myostatin and leptin levels did not significantly change.

**Table 3:** Between‐group comparison of differences during 3 month intervention period

			95% CI		
	β	SE	lower	upper	*P* value
**Body composition and functional assessment**					
BMI	0.13	0.09	−0.06	0.31	0.180
Waist circumference (cm)	−1.54	0.82	−3.15	0.07	0.061
Hip circumference (cm)	0.11	0.61	−1.09	1.31	0.861
RASM (kg/m^2^)	−0.03	0.05	−0.13	0.07	0.532
Male	0.12	0.10	−0.09	0.32	0.261
Female	−0.06	0.05	−0.16	0.04	0.265
Gait speed (m/s)	0.04	0.05	−0.06	0.14	0.436
6‐min walking distance (m)	23.83	8.65	6.88	40.79	0.006
5‐time sit‐to‐stand (s)	−0.87	0.37	−1.59	−0.15	0.017
Handgrip strength (kg)	0.62	0.54	−0.44	1.67	0.252
IPAQ (kcal)	445.09	231.87	−9.37	899.54	0.055
MNA	0.96	0.39	0.20	1.71	0.013
CESD	0.37	1.02	−1.63	2.37	0.715
**Laboratory data**					
Albumin (mg/dL)	0.02	0.04	−0.06	0.09	0.696
ALT (IU/mL)	−2.40	2.07	−6.44	1.66	0.248
Creatinine (mg/dL)	−0.02	0.01	−0.05	0.01	0.170
Fasting glucose (mg/dL)	0.89	2.54	−4.09	5.87	0.727
HOMA_IR	0.02	0.37	−0.71	0.74	0.965
Total cholesterol (mg/dL)	−8.30	4.85	−17.81	1.21	0.087
Triglyceride (mg/dL)	−15.01	6.54	−27.83	−2.20	0.022
HDL (mg/dL)	2.13	1.47	−0.74	5.00	0.146
LDL (mg/dL)	−9.23	3.96	−16.98	−1.47	0.020
CRP (mg/dL)	0.05	0.07	−0.08	0.18	0.475
DHEA‐S (μg/dL)	9.98	4.86	0.453	19.51	0.040
Leptin (pg/mL)	−1365.90	890.94	−3112.12	380.32	0.125
WBC (10^3^/uL)	−0.16	0.20	−0.55	0.23	0.427
Hb (g/dL)	0.12	0.14	−0.16	0.39	0.401
Myostatin (pg/mL)	−171.29	272.05	−704.50	361.91	0.529

ALT, alanine transaminase; BMI, body mass index; CCI, Charlson Co‐morbidity Index; CES‐D, Center for Epidemiological Studies Depression Scale; CRP, C‐reaction protein; DHEA‐S, dehydroepiandrosterone sulfate; Hb, haemoglobin; HDL, high‐density lipoprotein cholesterol; HOMA‐IR, homeostasis model assessment of insulin resistance; IPAQ, The International Physical Activity Questionnaire; LDL, low‐density lipoprotein cholesterol; MNA, Mini‐Nutritional Assessment; MoCA, Montreal Cognitive Assessment; RASM, relative skeletal muscle index; WBC, white blood cell.

## Discussion

This randomized controlled trial investigated the effects of protein‐enriched soup supplementation plus once weekly exercise on community‐dwelling middle‐aged and older individuals with inadequate dietary protein intake (1.0 g/kg/day), revealing significant improvements in muscle function, nutrition status, lipid metabolism, and DHEA‐S levels following 12 weeks of intervention. Several studies have demonstrated a synergic effect of protein supplementation on muscle anabolism and function after exercise in young and middle‐aged adults. However, on those with protein insufficiency, evidence supporting the combined effects were less robust and inconsistent. A systematic review exploring the concurrent influence of protein and amino acid supplementation in conjunction with exercise training on older individuals revealed that no additional benefit in physical performance attributed from nutrition supplement.^32^ Besides, the Vitality, Independence, and Vigour 2 study (VIVE2), examining multi‐nutritional supplementation (whey protein 20 g and Vitamin D 800 IU) alongside exercise training (3 times/week) for 6 months in mobility‐limited older adults revealed no further improvement with added nutritional supplementation in gait speed.^33^, ^34^ However, the current study showed significant improvement, which differed from the above‐mentioned studies. Previous studies primarily provided exercise frequency of three times a week, along with varying amounts of protein intake whether they are healthy or pre‐frail. Despite that exercising three times a week or more aligns with the guidelines of exercise for older adults, it is hard to achieve in daily life for healthy or frail older people. Therefore, our research design closely resembles the lifestyle and dietary habits of older individuals with inadequate protein intake, who may be at risk of pre‐frail or frail status. We incorporated a once‐a‐week group exercise training along with protein‐enriched soup. Our previous study demonstrated that maintaining sufficient dietary protein intake (>1.2 g protein/kg/day) without specific exercise still resulted in significant reductions in body weight, total body fat percentage, and hand grip strength among healthy community‐dwelling adults.^25^ The implications of these findings indicate that middle‐aged and older adults can enhance their body composition, muscle mass, muscle strength, and physical performance by ensuring sufficient protein intake, even in the absence of specific exercise interventions or with minimal exercise conducted only once a week. Besides, a previous cohort study conducted in Taiwan revealed that even minimal levels of physical activity were associated with a substantial reduction in mortality risk and an increase in life expectancy.^35^ Therefore, from the results of these studies, it is essential to prioritize adequate protein intake while carefully adjusting the exercise dose based on their health status and exercise capacity.

Besides, in the aging process, physiological changes related to lower sensory function (e.g., smell, taste, and vision), reduced swallowing ability, decreased bowel movements, and the accumulation of appetite‐inhibiting hormones such as cholecystokinin and leptin can result in inadequate protein intake, eventually leading to sarcopenia and frailty.^36^ A previous study demonstrated that protein‐enriched soup not only provided a palatable flavour for older adults but also improved protein intake.^37^ As a result, the incorporation of protein‐enriched soup in this study serves a dual purpose: It provides a diverse source of nutrients and has the potential to stimulate the appetite of older individuals, thus facilitating the attainment of sufficient intake levels.

In addition to enhancing physical fitness and muscle mass in older adults, protein supplementation has demonstrated its effectiveness in managing lipid profiles. Our study showed that middle‐aged and elderly individuals who previously had a suboptimal protein intake exhibited a notable 11% decrease in triglycerides and a 4% rise in HDL‐cholesterol when enhanced their diet with protein‐enriched soup for 3 months. In a meta‐analysis encompassing 22 studies, it was discerned that whey protein supplementation led to a reduction in triglycerides levels. However, the results concerning the impact on HDL‐C were inconclusive.^38^ The discrepancies in these findings primarily stem from differences in research design, methodologies, demographics of the study participants, and study duration. Distinguishing our study from others, our randomized controlled trial consisted of a relatively larger sample size, with older and at risk of frailty with insufficient daily protein intake, which prominently illustrate the effectiveness of nutritional enhancement for older adults experiencing malnutrition and vulnerability. Despite the lack of significant changes in body composition and daily activities during the intervention, our study found that the benefits of protein‐enriched soup supplementation plus weekly exercise on LDL‐C and triglyceride levels, indicating the potential of protein supplementation independent of exercise and body composition. Future endeavours in research necessitate more extensive analysis and exploration the benefit from protein intake and lipid metabolism.

In our research, we observed that DHEA, which is converted into active androgens or estrogens^39^ and experiences a decline with age, reaches only 10–20% of its original levels in individuals in their 70s and 80s.^40^ Notably, our study recorded a significant increase in serum DHEA‐S levels following the intervention. This potential mechanism aligns with the findings reported by Morgan et al., where protein consumption was positively associated with serum insulin‐like growth factor 1 (IGF‐1) levels in the middle‐aged and older population.^41^ Upon metabolism into active androgens or oestrogens, DHEA‐S exerts anabolic effects by enhancing the bioavailability of IGF‐1 in muscles, thereby aiding muscle growth and repair.^42^ Additionally, our research revealed that although the intervention group exhibited improvements in muscle health, intriguingly, there was no significant difference in the serum levels of myokines (myostatin) and leptin. This corresponds with our previous research, which indicated that serum myostatin levels are not suitable biomarkers for sarcopenia in relatively healthy older adults, a revelation that bears relevance to the current study.^43^ Hence, maintaining sufficient daily protein intake would be the prioritized to enhance muscle health and metabolic health, and even once weekly group exercise could potentiate the benefits through the anabolic effects, independent from the effects of myokines and adipokines.

Previous literature suggests that a combined intervention of nutrition and exercise may enhance muscle mass, muscle strength, endurance, and physical performance. However, in this trial, the improvements in muscle mass, muscle strength and physical performance, specifically gait speed, were not statistically significant. This could be attributed to the weekly exercise program, which, while more feasible for adherence among older individuals, may not confer comprehensive benefits. Further research is warranted to determine the optimal and feasible exercise intensity for promoting healthy aging among individuals with varying baseline conditions.

Notwithstanding the research efforts dedicated to this study, some limitations still persisted despite our best endeavours. First, the relatively short intervention period may impose limitations on assessing the potential long‐term benefits and whether there exists a ceiling effect for the intervention program. Second, although the participants exhibited good tolerance towards the protein‐enriched soup, it remains uncertain whether long‐term adherence to this specific dietary intervention would be feasible or sustainable among the older population. Thirdly, participants in this study exhibited a certain level of physical activities despite inadequate protein intake, indicating that their non‐sedentary lifestyle may partly account for the absence of improvements in body composition and other measured parameters. Fourth, the study constituted an open‐label intervention trial in which behavioural modifications could have influenced the efficacy of a protein‐enriched dietary and exercise regimen. Notably, the improvement in handgrip strength in the control group and MNA in intervention group were documented over the course of the 3 month study. These observations may be attributed to the health education potentially leading to lifestyle alterations during the study period. Last, in our study, there was no additional stratification of the intervention groups into those receiving only protein‐enriched soup and those participating in weekly exercise, we cannot definitively ascertain whether the observed beneficial effects are primarily attributable to exercise or nutritional supplementation. Future research is necessary to distinguish the individual roles of nutritional supplementation and exercise.

In summary, a 12‐week intervention involving protein‐enriched soup and once‐a‐week exercise resulted in significant improvements in physical function, nutrition status, lipid metabolism, and DHEA‐S levels. Further research is necessary to establish whether a saturation point exists for the observed beneficial effects and to evaluate the long‐term viability of sustaining the intervention's effects over an extended duration.

## Conflict of interest

The authors declare no conflict of interest.

## Supporting information


**Data S1.** The comparison of the items of Mini‐Nutritional Assessment (MNA)‐
