# Supplementary material for: Protein‐enriched soup and weekly exercise improve muscle health: A randomized trial in mid‐to‐old age with inadequate protein intake
Source: J Cachexia Sarcopenia Muscle. 2024 Apr 20;15(4):1348–57. doi: 10.1002/jcsm.13481 (PMC11294020; doi:10.1002/jcsm.13481)
Supplement: Supplementary file 1 — Data S1. The comparison of the items of Mini‐Nutritional Assessment (MNA)‐ [file JCSM-15-1348-s001.docx]

| **Supplementary 1. The comparison of the items of Mini-Nutritional Assessment (MNA)** | | | | |
| --- | --- | --- | --- | --- |
|  | Total | Intervention group | Placebo  group | p value |
|  | (N=97) | (N=47) | (N=50) |  |
| Has food intake declined over the past 3 months due to loss of appetite, digestive problems, chewing or swallowing difficulties? (no decrease in food intake) | 97(100%) | 47(100%) | 50(100%) | 1.000 |
| Weight loss during the last 3 months (weight loss between 1 and 3kg) | 20(20.6%) | 14(29.8%) | 6(12.0%) | 0.066 |
| Mobility (goes out) | 97(100%) | 47(100%) | 50(100%) | 1.000 |
| Has suffered psychological stress or acute disease in the past 3 months | 4(4.1%) | 3(6.4%) | 1(2.0%) | 0.278 |
| Neuropsychological problems (no psychological problems) | 97(100%) | 47(100%) | 50(100%) | 1.000 |
| Body Mass Index (BMI) |  |  |  | 0.736 |
| BMI<19 | 10(10.3%) | 6(12.8%) | 4(8.0%) |  |
| 19≤BMI≤21 | 16(16.5%) | 9(19.1%) | 7(14.0%) |  |
| 21≤BMI≤23 | 37(38.1%) | 17(36.2%) | 20(40.0%) |  |
| BMI>23 | 34(35.1%) | 15(31.9%) | 19(38.0%) |  |
| Lives independently (not in nursing home or hospital) | 97(100%) | 47(100%) | 50(100%) | 1.000 |
| Takes more than 3 prescription drugs per day | 20(20.6%) | 11(23.4%) | 9(18.0%) | 0.511 |
| Pressure sores or skin ulcers (no) | 97(100%) | 47(100%) | 50(100%) | 1.000 |
| How many full meals does the patient eat daily |  |  |  | 0.916 |
| 2 meals | 19(19.6%) | 9(19.1%) | 10(20.0%) |  |
| 3 meals | 78(80.4%) | 38(80.9%) | 40(80.0%) |  |
| Selected consumption markers for protein intake |  |  |  |  |
| -At least one serving of dairy products (milk, cheese,  yoghurt) per day | 40(41.2%) | 21(44.7%) | 19(38.0%) | 0.504 |
| -Two or more servings of legumes or eggs per week | 96(99.0%) | 47(100%) | 49(98.0%) | 0.330 |
| -Meat, fish or poultry every day | 89(91.8%) | 42(89.4%) | 47(94.0%) | 0.407 |
| Consumes two or more servings of fruit or vegetables per day? | 92(94.8%) | 45(95.7%) | 47(94.0%) | 0.698 |
| How much fluid (water, juice, coffee, tea, milk...) is consumed per day |  |  |  | 0.572 |
| 3 to 5 cups | 10(10.3%) | 4(8.5%) | 6(12.0%) |  |
| more than 5 cups | 87(89.7%) | 43(91.5%) | 44(88.0%) |  |
| Mode of feeding (self-fed without any problem) | 97(100%) | 47(100%) | 50(100%) | 1.000 |
| Self-view of nutritional status (= is uncertain of nutritional state) | 17(17.5%) | 9(19.1%) | 8(16.0%) | 0.684 |
| In comparison with other people of the same age, how does the patient consider his / her health status? |  |  |  | 0.322 |
| not as good | 11(11.3%) | 7(14.9%) | 4(8.0%) |  |
| does not know | 1(1.0%) | 0(0.0%) | 1(2.0%) |  |
| as good | 29(29.9%) | 11(23.4%) | 18(36.0%) |  |
| better | 56(57.7%) | 29(61.7%) | 27(54.0%) |  |
| Mid-arm circumference (MAC)≥ 22 cm | 93(95.9%) | 45(95.7%) | 48(96.0%) | 0.512 |
| Calf circumference ≥ 31 cm | 92(94.8%) | 45(95.7%) | 47(94.0%) | 0.698 |
